# Production and purification of staphylococcal nuclease in *Lactococcus lactis *using a new expression-secretion system and a pH-regulated mini-reactor

**DOI:** 10.1186/1475-2859-9-37

**Published:** 2010-05-21

**Authors:** Nicolas Trémillon, Nicolas Issaly, Julien Mozo, Thomas Duvignau, Hervé Ginisty, Eric Devic, Isabelle Poquet

**Affiliations:** 1 GTP-Technology, Immeuble Biostep, BP 48184, 31681 Labège Cedex, France; 2 INRA, UMR1319 Micalis (Microbiologie de l'Alimentation au service de la Santé), Domaine de Vilvert, Bâtiment 222, F-78352 Jouy-en-Josas cedex, France

## Abstract

**Background:**

Staphylococcal (or micrococcal) nuclease or thermonuclease (SNase or Nuc) is a naturally-secreted nucleic acid degrading enzyme that participates in *Staphylococcus aureus *spread in the infected host. Purified Nuc protein can be used as an exogenous reagent to clear cellular extracts and improve protein purification. Here, a recombinant form of Nuc was produced and secreted in a Gram-positive host, *Lactococcus lactis*, and purified from the culture medium.

**Results:**

The gene segment corresponding to the *S. aureus *nuclease without its signal peptide was cloned in an expression-secretion vector. It was then fused to a lactococcal sequence encoding a signal peptide, and expressed under the control of a lactococcal promoter that is inducible by zinc starvation. An *L. lactis *subsp *cremoris *model strain (MG1363) transformed with the resulting plasmid was grown in either of two media (GM17v and CDM) that are free of animal compounds, allowing GMP (Good Manufacturing Practice) production. Induction conditions (concentration of the metal chelator EDTA and timing of addition) in small-scale pH-regulated fermentors were optimized using LacMF (Lactis Multi-Fermentor), a home-made parallel fermentation control system able to monitor 12 reactors simultaneously. Large amounts of recombinant Nuc (rNuc) were produced and secreted in both media, and rNuc was purified from GM17v medium in a single-step procedure.

**Conclusions:**

In *L. lactis*, rNuc production and secretion were optimal after induction by 0.5 mM EDTA in small scale (200 mL) GM17v exponential phase cultures (at an OD_600 _of 2), leading to a maximal protein yield of 210 mg per L of culture medium. Purified rNuc was highly active, displaying a specific activity of 2000 U/mg.

## Background

*L. lactis *which is widely used as a starter in dairy industries, is also an efficient cell factory for the production and secretion of proteins [1]. i) Proteins produced in this species, which is considered as safe by virtue of its longtime human consumption, should obtain the GRAS (Generally Recognized As Safe) status and be suitable for therapeutical or vaccine applications [1], in contrast to proteins produced in *Escherichia coli *from which endotoxin (LPS) has to be removed [2]. ii) In this Gram^+ ^species, the secretion of any heterologous protein fused to an appropriate signal peptide can be easily and efficiently driven by the general export (Sec) pathway [1], thus avoiding potential intra-cellular toxicity and/or misfolding. iii) In this species, an extracellular protease-free strain (without the unique surface protease HtrA [3]) is a useful host to avoid protein degradation [4], whereas in *Bacillus subtilis*, not all extracellular proteases have been inactivated to date (4 active surface proteases, including the 3 HtrA family members, are remaining in the available mutant strains) [5,6]. iv) Only one major protein, Usp45, is secreted in significant amounts into the medium [7], thus facilitating downstream purification steps. v) Finally, setting up conditions for protein production in large fermentors should be easy as culture scale-up is linear [8].

In this context, several gene expression systems have been developed for *L. lactis*: i) NICE, the most widely used system based on the P_*nis*__A _promoter and *nisRK *two-component regulatory system, is induced by nisin [9]; ii) P170, which is regulated by RcfB, is induced by lactic acid, in particular during the transition to the stationary phase of growth where the pH is low due to lactate accumulation [10,11] (SM Madsen, personal communication), and iii) P_Zn _is tightly regulated by the ZitR repressor in response to extra-cellular Zn^2+ ^levels: it is repressed in a wide concentration range from repletion to toxicity, and induced by starvation [12-14] (Daniel Llull, Olivier Son, Nicolas Trémillon, Sandrine Blanié, Julien Briffotaux, Sébastien Blugeon, Eric Morello, Hélène Rogniaux, Olivier Danot, and Isabelle Poquet: ZitR, a prototype of a new class of zinc responsive repressors in *Streptococcaceae*, submitted). As an expression system, ZitR-regulated P_Zn _should allow the repression of a potentially toxic heterologous ORF in the presence of Zn^2+ ^(e. g. in a rich medium), and once a sufficient amount of biomass has been obtained, its induction by addition of a chelator agent (e. g. EDTA) [12,14]. To allow protein secretion, expression systems have been combined with several signal peptides: i) that of Usp45 [1,7] ii) that of Exp4 [13,15,16], and iii) several optimized signal peptides (SP310 series) [17,18].

Finally, with the use of all available tools, *L. lactis *has proved to be an efficient host for the production and secretion of proteins of medical interest, generally in flasks for laboratory studies [1], but also in small scale (1L) fermentors [17]. Recently, the lysostaphin from *Staphylococcus simulans *biovar staphylolyticus was successfully produced at the industrial scale (3000 L) in *L. lactis *using the NICE system. Surprisingly though, this naturally-secreted protein was produced as a recombinant signal peptide-free form that had to be purified from the lactococcal cell extract [8,19].

In the present study, the efficiency of *L. lactis *as a host for heterologous protein production and secretion, and the ease of protein purification from a lactococcal culture medium were evaluated using the staphylococcal nuclease Nuc [20,21] as a model protein of biotechnological and commercial interest. Nuc is a robust exo- and endo-5'-phosphodiesterase (EC 3.1.31.1) active against both DNA and RNA [22-24]. It can be used for RNA sequencing [25] and in several applications where nucleic acid removal is desired, like reduction of the viscosity of a cell lysate, improvement of protein purification, and development of *in vitro *translation systems [26-28]. In *S. aureus*, Nuc participates in the spread of the bacterial cells in the infected host [29] as a naturally-secreted enzyme: cleavage of the precursor signal peptide leads to the secretion of the pro-peptide form (NucB) that is processed to the mature form (NucA) [30]. Different forms of Nuc protein have been produced in several species: the native wild-type form, in *Bacillus subtilis *[31], *Corynebacterium glutamicum *[32] and *L. lactis *[33], and recombinant forms fused to different signal peptides, in *E. coli *[26,34] and in *L. lactis *[16,35] where NucB processing to NucA was found to require HtrA protease [3,36].

In this study, a recombinant Nuc form (rNuc) was successfully produced and secreted in *L. lactis *using a recently developed expression-secretion system [13] and small-scale pH-regulated reactors. An active rNuc protein could be purified in a single step from the culture medium, indicating that secretion is a good method for facilitating the purification of a heterologous protein produced in *L. lactis*.

## Results

### Expression-secretion vector

**Figure 1:**
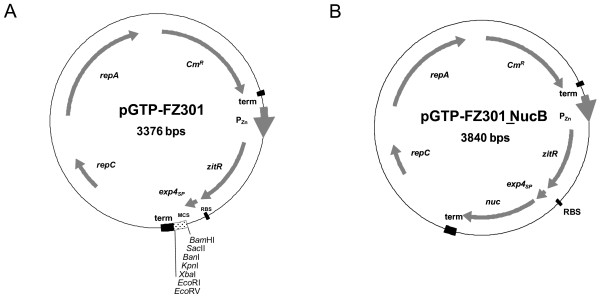
**Expression-secretion vector used for rNuc production**. (A) pGTP_FZ_301 allows any open reading frame to be cloned as a translational fusion to Exp4 signal peptide coding sequence, and expressed as an operon to *zitR *under the control of P_Zn _promoter. (B) pGTP_FZ_301_NucB results from the cloning of a recombinant truncated form of *S. aureus nuc *ORF (coding for NucB) into pGTP_FZ_301. NucB, the secreted mature form of staphylococcal Nuc (after signal peptide cleavage) is fused in frame to Exp4 signal peptide, leading to a recombinant precursor that is secreted as rNuc. MCS: Multi-Cloning Site; RBS: Ribosome Binding Site; term: terminator

pGTP_FZ301 (Figure 1A) is an expression and secretion vector for *L. lactis *that is derived from pLB145 [13]. pGTP_FZ301 enables any ORF to be cloned as a translational fusion to the lactococcal sequence encoding Exp4 signal peptide [15,16], and the fusion and *zitR *constitute an operon under the control of the P_Zn _promoter [12]. The cloning of *nucB *ORF (encoding NucB form) into pGTP_FZ301 resulted in pGTP_FZ301_NucB, and led to the production of a recombinant precursor that is secreted as rNuc (Figure 1B). This recombinant protein was designed because the wild-type Nuc precursor which bears an atypical signal peptide is not efficiently secreted in *L. lactis *[33], in contrast to a fusion between NucB and a lactococcal signal peptide [35].

### Optimization of induction conditions

**Figure 2:**
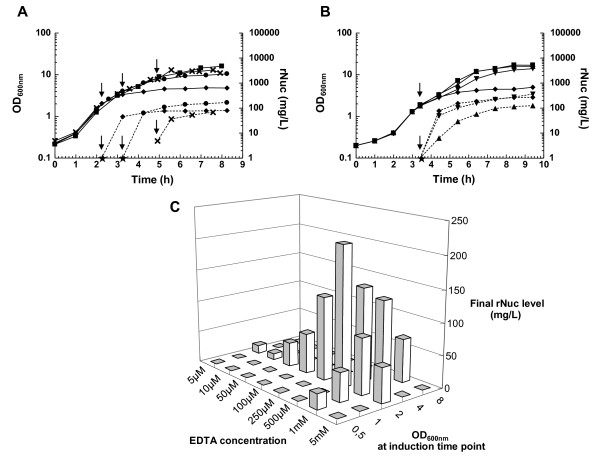
**Optimization of induction conditions for maximal rNuc production**. Cultures of strain 918 grown in GM17v using LacMF were induced by the addition of EDTA at different time points (A) or concentrations (B) or both (C). In (A) and (B), both bacterial growth (solid lines) and rNuc concentration into the medium (dotted lines, calculated by comparison to a quantified standard on stained gels) are shown as a function of time (↓ indicates EDTA addition time point). As before induction, rNuc level was undetectable for mid-exponential phase cultures (at OD_600 _2 and 4), an arbitrary value (1 mg/L, corresponding to the sensitivity threshold of our staining method) is indicated (black star). (A) rNuc levels as a function of time after induction of a culture at different growth stages by highly concentrated EDTA. 1 mM EDTA was added or not (black square) to parallel cultures at different OD_600_, i. e. 2 (black diamond), 4 (black circle) or 8 (X). (B) rNuc level as a function of time after induction of an exponential phase culture by EDTA at different concentrations. Cultures at OD_600 _2 were induced or not (black square) by EDTA at various concentrations: 100 μM (black triangle), 500 μM (upside down black triangle) or 1 mM (black diamond). (C) Final rNuc level as a function of both induction OD_600 _and EDTA concentration. Final rNuc concentration in the medium after prolonged growth (8 h-9 h of culture) is shown.

For rNuc production in *L. lactis *subsp *cremoris *strain 918 [MG1363(pGTP_FZ301_NucB)], a new medium was developed. This rich medium is free of animal compounds (GM17v), which could prove useful for the production of proteins that must be devoid of any potentially pathogenic contaminant, such as therapeutic proteins. In pH-regulated cultures using GM17v, a final OD_600 _of 15-16 could be reached (Figure 2).

The induction parameters of GM17v cultures were optimized taking previously published data about the lactococcal fermentation process [19] and induction conditions of ZitR-regulated P_Zn _in other media ([12] and data not shown) into account. Optimization was achieved using LacMF, a parallel fermentation control system able to monitor 12 mini-reactors simultaneously (Additional file 1, Figure S1).

To determine the P_Zn _induction conditions in GM17v, different concentrations of the metal chelator EDTA were added to cultures in different growth phases. Both cell growth and rNuc secretion in the medium were monitored (Figure 2). Before induction, the rNuc protein was undetectable in mid-exponential phase cultures (for uninduced cells at an OD_600 _of 2 or 4, data not shown and Figures 2A and 2B), and present in low amounts for late-exponential/stationary phase cultures (5 mg/L for uninduced cells at an OD_600 _of 8, Figure 2A). This result suggested that the P_Zn _promoter was first repressed and then progressively induced during growth in GM17v medium, probably because extra-cellular Zn^2+ ^became depleted, as previously observed in another rich medium, GM17 (data not shown), and in a chemically defined medium [12] (Daniel Llull, Olivier Son, Nicolas Trémillon, Sandrine Blanié, Julien Briffotaux, Sébastien Blugeon, Eric Morello, Hélène Rogniaux, Olivier Danot, and Isabelle Poquet: ZitR, a prototype of a new class of zinc responsive repressors in *Streptococcaceae*, submitted).

When added at 1 mM at an OD_600 _of 2 (Figure 2A) or below (data not shown), EDTA impaired growth which stopped as early as one generation after addition. When 1 mM EDTA was added at an OD_600 _of 4 or 8, the growth impairment seemed to be weaker, probably because late or post-exponential phase cells could almost reach the growth plateau after exposure to the inducer (Figure 2A). The time of induction had a significant effect on the level of rNuc in the medium. Induction before the OD_600 _reached 2 severely impaired growth and consequently low rNuc levels were obtained (data not shown). In contrast, when EDTA was added at an OD_600 _of 2 or above, rNuc accumulated rapidly within the first hour of induction, and a high final level of between 100 and 200 mg/L was reached. The optimum of 200 mg/L was obtained for induction at OD_600 _4 (Figure 2A).

In a second phase of optimization, different concentrations of EDTA were added at an OD_600 _of 2 (Figure 2B). Whereas EDTA at 1 mM (Figure 2A) or above (data not shown) severely impaired growth, little (a slightly reduced OD_600 _at the plateau) or no growth defect was observed for 500 μM or 100 μM EDTA (Figure 2B). rNuc production in the medium significantly varied with the inducer concentration. After induction with 100 μM EDTA, the rNuc level followed growth and reached its maximum only at the growth plateau, whereas higher EDTA concentrations led a drastic rNuc accumulation within the first hour of induction (Figure 2B) followed by a slight further augmentation afterwards. Interestingly, the fast kinetics of accumulation was not related to a growth defect as it could be observed for either 500 μM EDTA (Figure 2B) or 1 mM EDTA (Figure 2A). 500 μM was found to be the optimal inducer concentration when added at OD_600 _2 and allowed rNuc production to reach a level of 200 mg/L.

As these results indicated that (i) both time of induction (OD_600_) and EDTA concentration were important parameters for rNuc production, and that (ii) rNuc continued accumulating untill the growth plateau was reached (albeit sometimes slowly), the combined effect of both parameters on final rNuc levels after prolonged cultures was monitored (Figure 2C). rNuc increased in direct proportion to the EDTA concentration up to 500 μM. Above 1 mM EDTA, rNuc production did not improve regardless of the OD_600 _at which EDTA was added (Figure 2C). The maximal concentration of rNuc in the medium, 210 mg/L, was obtained when 500 μM EDTA was added to cells at an OD_600 _of 2 (Figures 2B and 2C), thus defining the optimal conditions for rNuc production and secretion in lactococcal cultures grown in GM17v.

**Figure 3:**
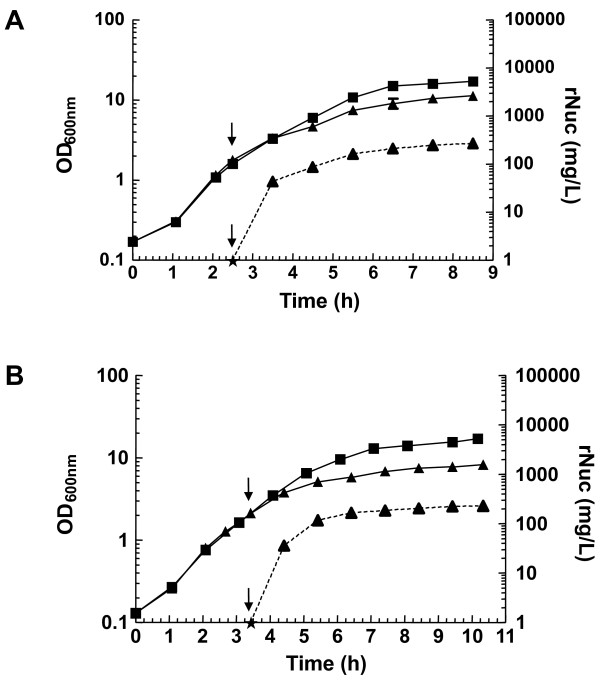
**rNuc production as a function of the culture medium**. Strain 918 was grown in either GM17v (A) or CDM (B) till an OD_600 _of 2, and induced or not (black square) by addition of 500 μM EDTA (black triangle). Culture growth and rNuc concentration in the medium are indicated by solid or dotted lines respectively. The induction time point and undetectable levels of rNuc (<1 mg/L, see Figure 2 Legend) are respectively indicated by ↓ and (black star) symbols.

In a second set of experiments, two media, the previously-used rich medium, GM17v, and a chemically defined medium, CDM [37], were compared. Interestingly, the induction conditions optimised for GM17v medium were applicable to CDM and allowed production of rNuc to comparable levels (Figure 3).

### One-step Purification and nuclease activity

**Figure 4:**
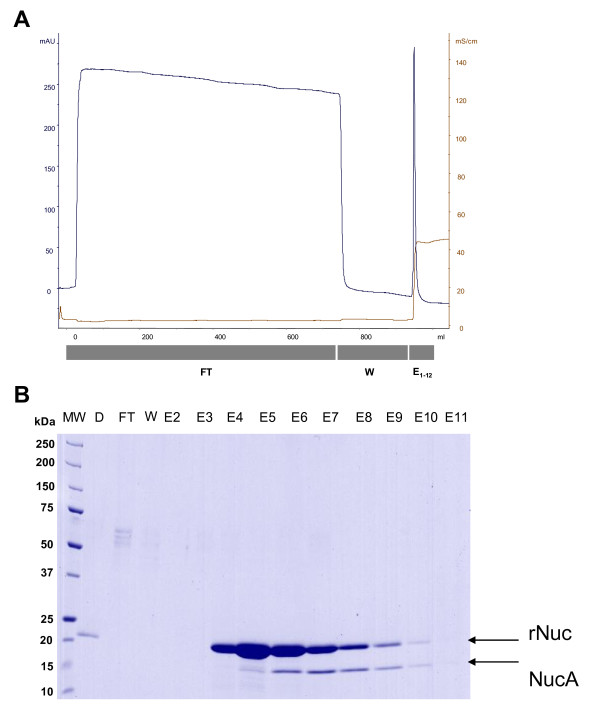
**Purification of secreted rNuc protein by cationic exchange chromatography**. (A) Ion exchange chromatogram for rNuc purified from the supernatant of strain 918 grown in GM17v. NaCl concentration (brown line) and absorption at 280 nm (blue line) are shown. Fractions analysed by SDS-PAGE are indicated by grey bars underneath. (B) SDS-PAGE analysis of different fractions (E2-11) of the cationic exchange chromatography stained by Coomassie brilliant Blue. MW: Molecular Weight Marker, D: Diluted culture supernatant, FT: Flow Through, W: Washing, E1-12: Elution fractions number 1-12. Secreted rNuc protein (after signal peptide cleavage by signal peptidase) and the mature NucA form (resulting from pro-peptide processing by HtrA surface protease, as previously observed [3]), are indicated by arrows.

rNuc produced and secreted by strain 918 at the highest level (after induction of cells grown in GM17v to an OD_600 _of 2 by 500 μM EDTA) was purified from the culture medium using a simple, previously-described single step procedure [38]. This procedure, based on cation exchange chromatography, excludes Usp45, the main secreted lactococcal protein [7] (Figure 4). Approximately 85% of rNuc could be recovered from the culture supernatant by this method (data not shown), and finally, ultrapure rNuc (> 99%) concentrated at 115 mg/L (as determined by SDS-PAGE analysis using Bovine Serum Albumin (BSA) as a standard) was obtained. Nuclease activity on denatured DNA was assayed as previously described [22,23], showing that purified rNuc exhibited a high specific enzymatic activity of 2000 U/mg (for comparison, this is 7-20 times higher than a marketed Nuc protein [39]).

## Discussion

To determine the conditions for the use of *L. lactis *to produce a secreted heterologous protein, we chose the staphylococcal nuclease, a protein used in the field of biotechnology. This nucleic acid degrading enzyme can be used in several applications, particularly protein purification, by reducing the viscosity of a cell lysate [26,27] and the nucleic acid contamination of the protein which, in the specific case of therapeutic proteins, is required by FDA to be less than 100 pg per dose [40].

A new system for the production of recombinant proteins was developed. As a cell factory, we used *L. lactis *grown in either a new, rich and animal compound-free medium (GM17v) or a chemically defined medium (CDM). The heterologous ORF was cloned in pGTP_FZ301 vector under the control of P_Zn _and fused to the sequence encoding the Exp4 signal peptide, and its expression was induced by the chelator EDTA. The whole production-secretion system should be fully compatible with regulatory restrictions in bioproduction for human therapeutics, and the proteins produced with it should be considered as GRAS products. Alternatively, *L. lactis *grown in CDM medium might also be an interesting host system for specific amino acid labelling (C^13^, N^15^, seleno-methionine or seleno-cysteine) and protein structural studies through NMR or X-Ray crystallography.

For cell growth, a pH-regulated batch fermentation process was used. Using a parallel fermentation control system (LacMF), the pH value was maintained at the set point by the addition of a mild alkaline agent, NH_4_OH. It was previously shown that pH neutralization leads to prolonged exponential growth of *L. lactis *and increases the final cell density about fivefold compared with pH-unregulated batch culture fermentation [17,19]. Indeed, the biomass of pH-regulated cultures reached high levels (final OD_600 _of around 15-16, Figures 2 and 3). NH_4_OH addition also maintains lactic acid in its dissociated lactate form which is less-toxic [41] even though at high concentrations, it also slows growth [42]. To further increase the productivity of the expression system used here, lactate [43,44] should be continuously extracted using continuous perfusion [45] (unpublished results) and electro-dialysis (i. e. REED) [46]. The latter technology with lactococcal expression system P170, allowed to reach protein yields in the gram per liter range [47]. Medium composition could also be optimized, as the addition of nitrogen and carbon sources was previously found to significantly increase protein production in *L. lactis *grown in a rich medium [19].

LacMF was a useful tool to optimize EDTA induction in *L. lactis*. The best way to induce the P_Zn _promoter was to add a non-toxic concentration of EDTA to an exponential phase culture, in agreement with what had previously been observed in another medium [12]. Induction in the exponential phase should maximize the effective production period before the stationary phase and toxic lactate accumulation.

In the absence of induction, rNuc is first undetectable when produced by exponentially growing cells, suggesting that its expression is repressed, and then weakly induced in late-exponential/stationary phase cells (Figures 2A and 2B), as previously observed in another medium [12] (Daniel Llull, Olivier Son, Nicolas Trémillon, Sandrine Blanié, Julien Briffotaux, Sébastien Blugeon, Eric Morello, Hélène Rogniaux, Olivier Danot, and Isabelle Poquet: ZitR, a prototype of a new class of zinc responsive repressors in *Streptococcaceae*, submitted). This growth-phase dependent regulation suggests that free Zn^2+^, initially present in the medium at repression levels, could become exhausted or unavailable during growth, thus leading to progressive P_Zn _induction [12] (Daniel Llull, Olivier Son, Nicolas Trémillon, Sandrine Blanié, Julien Briffotaux, Sébastien Blugeon, Eric Morello, Hélène Rogniaux, Olivier Danot, and Isabelle Poquet: ZitR, a prototype of a new class of zinc responsive repressors in *Streptococcaceae*, submitted). This could also explain the differences in rNuc accumulation kinetics according to the inducer concentration. When induced at high EDTA concentration (at and above 500 μM), rNuc rapidly accumulates (within 1 h) almost to its maximal level, whereas at 100 μM EDTA, accumulation is much slower and goes on for a longer time (Figure 2B). Interestingly, rNuc levels of exponentially growing cells induced by 100 μM EDTA for 1 h (Figure 2B) and of uninduced cells at OD_600_=8 (Figure 2A) are similar, suggesting that 100 μM EDTA only has a weak inducing effect, in agreement with the observation that it has only a slight, if any, effect on cell growth (Figure 2B). The final rNuc level after several hours of induction by 100 μM EDTA (Figure 2B) might result from an increased production rate due to a higher biomass in a medium progressively depleted of Zn^2+^.

The EDTA concentration for optimal induction is significantly higher (more than 10 times) in both GM17v and CDM medium than in SA, the medium we previously used [12]. This is not surprising, as CDM and SA media are highly different, notably for their micro-nutrient composition (for example Mn^2+ ^and Cu^2+ ^are not added to CDM in contrast to SA), and in particular for Zn^2+ ^content, which is much higher (3 orders of magnitude) in CDM than in SA [37,48]. Meanwhile, in both CDM and GM17v medium, the same optimal inducer concentration leads to a similar level of rNuc in the medium. As P_Zn _expression level was recently found to be inversely correlated to extra-cellular Zn^2+ ^concentration (Daniel Llull, Olivier Son, Nicolas Trémillon, Sandrine Blanié, Julien Briffotaux, Sébastien Blugeon, Eric Morello, Hélène Rogniaux, Olivier Danot, and Isabelle Poquet: ZitR, a prototype of a new class of zinc responsive repressors in *Streptococcaceae*, submitted), this indicated that GM17v and CDM media had similar Zn^2+ ^contents.

Induction by EDTA at high concentration (1 mM) in GM17v and CDM led to a growth defect of strain 918 (Figures 2A, 2B and 3A). However, this was not due to the toxicity of EDTA *per se*, because of chelation of divalent cations to detrimental levels and cell starvation, as 1 mM EDTA specifically affected strain 918 but not the parental strain MG1363 (under identical conditions in the absence of antibiotic selection; data not shown). The specific growth defect of strain 918 thus seems to result from the presence of the pGTP_FZ301_NucB plasmid, and it is tempting to speculate that it could be due to the metabolic burden of secreted rNuc overproduction. In *B. subtilis*, a similar inducer-dependent growth defect has already been described for a strain over-expressing an heterologous secreted protein: it was associated to a secretion stress that was shown to lead to an adaptative cell response including the induction of the CssRS regulon [49-51]. This study should help define the conditions for hitherto unexamined secretion stress in *L. lactis*.

In this study, under optimal induction conditions, an rNuc production yield of 210 mg/L was reached. This yield is comparable to the ones previously obtained for staphylococcal nuclease forms produced in pH-regulated fermentative cultures of *L. lactis *using inducible P170 promoter [52] or the constitutive P_*usp45 *_promoter [38]. The efficiencies of the ZitR-regulated P_Zn _and NICE expression systems were found to be comparable under classical conditions [12]. All these comparisons indicate that pGTP_FZ301 is an efficient tool and a useful alternative expression-secretion system in *L. lactis*.

rNuc could be purified from a lactococcal culture medium in a single-step process. As *L. lactis *secretes few native proteins, heterologous protein secretion greatly simplifies downstream processing and purification. Indeed, after a single purification using cationic exchange resin, 85% of the rNuc protein was recovered pure at 99% and fully active. For comparison, the cytoplasmic recombinant form of lysostaphin produced in *L. lactis *using the NICE expression system was recovered to 80%, and was only 90% pure after three steps of cation exchange chromatography [19]. Similarly, the staphylococcal nuclease R produced by *E. coli *required two steps of metal chelating affinity chromatography to be purified from a cell extract [53].Thus secretion in *L. lactis *appears to simplify the downstream purification process.

## Conclusions

This study for the first time describes the use of the promoter P_Zn _in pH-regulated mini-reactors. Optimization of induction conditions for nuclease production was rapidly achieved with the use of the pH controller LacMF and allowed to reach a yield of 210 mg/L. Nuclease produced by *L. lacti*s was purified from the culture supernatant, providing a highly pure and active enzyme that should be useful for removing RNA and DNA from cell extracts. The fermentation, production and purification processes that were set up for the production of staphylococcal nuclease in *L. lactis *proved to be competitive, and they should be used in the future for different heterologous proteins, like proteins of therapeutical value.

## Methods

### Bacterial strains and standard culture conditions

*E. coli *NEB 5-α (New England Biolabs, Ipswich, MA) was grown at 37°C with 200-250 rpm shaking in reconstituted Luria Bertani (LB) broth: 1% tryptone (Sigma, St Louis MO), 5% yeast extract (Fluka, St Louis MO), 1% NaCl (Fluka) resuspended in pure water, supplemented with ampicillin at 100 μg/mL (Sigma) when necessary. Solid media were prepared by adding technical agar (Invitrogen, Paisley UK) at a final concentration of 1.5% w/v. *L. lactis *MG1363 strain [54] and strain 918 [i. e. MG1363(pGTP_FZ301_NucB)] were routinely grown at 30°C without shaking in rich M17 (Fluka) supplemented with 1% glucose and with chloramphenicol 5 μg/mL (Sigma) for plasmid selection in the case of strain 918.

### A parallel fermentation system able to monitor 12 mini-reactors

LacMF (Additional file 1, Figure S1) is a proportional, integrative and derivative (PID) controller that allows continuous pH monitoring and control of 12 simultaneous mini-reactors of 50 mL-1 L. pH is maintained at the set point by adding 30% v/v NH_4_OH (Fluka) to the different cultures, using a pump with twelve solenoid valves (Additional file 1, Figure S1) that open sequentially and for limited times. NH_4_OH neutralizes lactate produced by fermentation, thus impeding medium acidification and leading to prolonged exponential growth and a higher biomass. Cultures are maintained at 30°C and continuously homogeneized by a magnetic stirrer (with 100-150 rpm agitation). LacMF system allowed optimization to be performed quickly, within two weeks and only 4 rounds of 12 independent experiments.

### Fermentation and induction conditions in mini-reactors

Strain 918 was inoculated to an initial OD_600 _of 0.2 in 200 mL mini reactors and grown under standard fermentation conditions: at 30°C and pH 6.5 using LacMF. Two different media: a chemically defined medium, CDM [37], or a rich medium free of animal compounds, GM17v, were used. GM17v contains 1% vegetable extract (Fluka), 0.25% yeast extract (Fluka), 0.05% L-ascorbic acid (Sigma), 1.9% Sodium glycero-phosphate (Sigma), 0.05% MgSO_4 _(Fluka) and 5% glucose. When necessary, EDTA 0.5 M w/v (Sigma) was added to reach the indicated final concentrations and induce rNuc expression. Samples were harvested every hour to monitor bacterial growth by spectrophotometrically measuring absorbance at 600 nm.

### Plasmids

**Figure 5:**
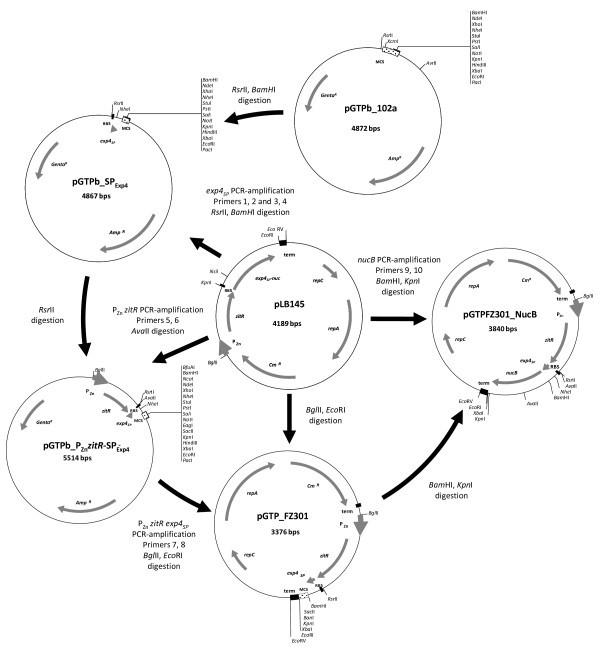
**Cloning strategy**. The construction of the various plasmids used in this study is shown (see the Plasmids paragraph in Methods section for further details).

Plasmids used in this study are listed in Table 1 and cloning strategy is shown in Figure 5. Sequence coding for Exp4 signal peptide (SP_Exp4_) was amplified from pLB145 [13] in a two-step PCR: using primers 1 and 2 (see Table 2 for primer sequences) for 5 cycles, and then primers 3 and 4 for 25 cycles. PCR product digested by *Rsr*II and *Bam*HI was cloned into pGTPb_102a cloning vector digested by the same enzymes, and the ligation was used to transform *E. coli *NEB 5-α. pGTPb_SP_Exp4 _from an ampicillin resistant clone was verified by digestion and sequencing. P_Zn _*zitR *expression cassette was PCR-amplified from pLB145 using primers 5 and 6. PCR product digested by *Ava*II was cloned into pGTPb_102a_SP_Exp4 _previously digested by *Rsr*II and dephosphorylated. Ligation reaction was transformed into NEB 5-α competent cells. pGTPb_P_Zn _*zitR-*SP_Exp4 _from an ampicillin-resistant clone was verified by digestion and sequencing, revealing three silent mutations in *zitR *sequence. P_Zn _*zitR *SP_Exp4 _expression and secretion cassette was PCR-amplified from pGTPb_P_Zn _*zitR-*SP_Exp4 _using primers 7 and 8. PCR product digested by *Bgl*II and *Eco*RI was ligated into pLB145 previously digested by the same enzymes, and the ligation was introduced into *L. lactis *MG1363 competent cells. pGTP_FZ301 from a chloramphenicol-resistant clone was verified by digestion and sequencing. *nucB *ORF that encodes the mature secreted part (NucB or pro-Nuc) of staphylococcal nuclease after signal peptide cleavage [30] was PCR-amplified from pLB145 [13] using primers 9 and 10. PCR product was digested by *Bam*HI and *Kpn*I, cloned into pGTP_FZ301 previously digested by *Bam*HI and *Kpn*I, and ligation was used to transform MG1363. pGTP_FZ301_NucB plasmid extracted from a chloramphenicol resistant clone was verified by digestion and sequencing.

**Table 1:** Plasmids used in this study.

Name	Characteristics	Reference
pLB145	Cm^R^, pWV01 derivative carrying *exp4*_SP_*-nucB *gene fusion under the control of ZitR-regulated P_Zn _promoter	[13]
pGTPb_102a	Amp^R^, pFastBacHta (Invitrogen Paisley UK) derivative carrying an ORF for HFFT tag (His Flag FoldFold Flag Tev) [56-58]	This study
pGTPb_SP_Exp4_	Amp^R^, pGTPb_102a derivative where the ORF for Exp4 signal peptide (SP_Exp4_) has been cloned	This study
pGTPb_P_Zn _*zitR-*SP_Exp4_	Amp^R^, pGTPb_SP_Exp4 _derivative where *zitR *is in operon with the ORF for SP_Exp4 _under P_Zn _control	This study
pGTP_FZ301	Cm^R^, pLB145 derivative where a multi-cloning site has been cloned after the ORF for SP_Exp4 _(in operon with *zitR *under P_Zn _control)	This study
pGTP_FZ301_NucB	Cm^R^, pGTP_FZ301 derivative where *nucB *ORF has been cloned leading to a gene fusion encoding SP_Exp4_-NucB precursor and expressed under the control of ZitR-regulated P_Zn _promoter	This study

**Table 2:** Oligonucleotides used in this study.

Name	Sequence
1	5' ACTCGGTCCGTACCTTAAGGAGATATAAAAATGA 3'
2	5' TTTTTTGGATCCAAACCTGCCAGTATCATCAGCAAATACAACGGCT 3'
3	5' GAAAAAAACTCGGTCCGTACCTTAAGGAGA 3'
4	5' GTTTTTTTTTTGGATCCAAACCTGCCAGT 3'
5	5' GATATATATATGGTCCAGATCTTTGATCAAGGATCTGTC 3'
6	5' TCCTTAAGGTACGGACCGTCTTCATCGAAACTCTTCAGT 3'
7	5' AAAATGATAACCATCTCGCAA 3'
8	5' CTACAAATGTGGTATGGCTGAT 3'
9	5' TTTAAATTTAGGATCCGCATCACAAACAGATAACGG 3'
10	5' TATATATATAGGTACCTTATTGACCTGAATCAGCGT 3'

Restriction enzymes, T4 DNA ligase and Antartic phosphatase (New england Biolabs, Ipswich, MA), high fidelity Phusion™ DNA polymerase (Finnzymes, Espoo, Finland) were used according to recommendations. DNA purification kits were purchased from Macherey-Nagel (Düren, Deutschland). Sequencing was performed by Genome express (Meylan, France).

### SDS-PAGE and protein quantification

Supernatants were separated from cell samples by centrifugation and stored at -20°C. Protein samples were analysed by SDS-PAGE using gradient (7.5-16.8%) gels and Precision Plus Protein Standards was used for molecular weight estimation (Biorad, California, USA). Commercial BSA (Sigma) was used as a standard for protein quantification. Gels were stained by Coomassie blue G250 (Biorad), scanned (GS800 Calibrated densitometer, Biorad) and analyzed using Image Quant (Amersham Biosciences, Uppsala Sweden).

### Nuclease purification

Culture supernatant was filtrated on 0.22 μm membrane, diluted 15-fold in ultrapure water and loaded on a 10 mL SP sepharose column (GE healthcare, Hillerod, Denmark). All purification steps were performed on an AKTA purifier (GE healthcare). Column was washed with 20 volumes of washing buffer (15 mM sodium phosphate buffer, 15 mM NaCl, pH 7.5). Nuclease was eluted by 10 volumes of elution buffer I (15 mM sodium phosphate buffer, 500 mM NaCl, pH 7.5) and automatically collected in fractions of 2.5 mL using FRAC910 (GE Healthcare). Elution fractions were checked by SDS-PAGE, pooled together and dialysed four times using a Spectra POR dialysis membrane with a cut-off of 3.5 kDa (Spectrum, Rancho Dominguez, CA) against 500 mL of dialysis buffer (20 mM Tris buffer, 100 mM NaCl, 2 mM EDTA, pH.7.5). Nuclease concentration was measured by densitometry and the purified protein was stored at -20°C in storage buffer (10 mM Tris buffer,, 50 mM NaCl, 1 mM EDTA, 50% glycerol, pH 7.5).

### Nuclease assay

Nuclease activity was assayed using a modified version of a previously described method [22]. 10 μL of purified nuclease protein was incubated in 500 μL of reaction buffer (25 mM Tris-HCl, 10 mM CaCl_2_, 0.01% BSA (w/v), 0.1% Salmon sperm DNA pH 8.8) at 37°C for 30 min, and the reaction was stopped by addition of 500 μL of 4% (v/v) perchloric acid and left for 15 min on ice. In negative controls, purified nuclease protein was added after the addition of perchloric acid. Acid-insoluble nucleic acids were sedimented by centrifugation for 10 min at 15000 g at 4°C. DNA hydrolysis was determined by spectrophotometrically measuring the absorbance of acid soluble polynucleotides at 260 nm (One unit is defined as producing 1 μmole of acid soluble polynucleotides from DNA per minute at pH 8.8 and 37°C [55]).

## Competing interests

The authors declare that they have no competing interests.

## Authors' contributions

NT designed and supervised experiments and drafted the manuscript. NI initiated the experiments. JM and TD respectively supervised purification and fermentation experiments. HG, ED and IP defined the strategy and supervised the project. HG and ED helped to draft manuscript, and IP edited the manuscript. IP supervised the entire PhD project of NT. All authors read and approved the final manuscript.

## Supplementary Material

Additional file 1 **LacMF, a parallel fermentation control system**. 12 lactococcal cultures in mini-reactors of 200 mL can be made in parallel. They are maintained at 30°C and continously homogeneized by a magnetic stirrer ༉), and pH is controlled by supplying a neutralizing agent, NH_4_OH, via a proportional, integrative and derivative (PID) controller. NH_4_OH is added to the mini-reactors by a pump (⇐) with twelve solenoid valves (⇓). Click here for file
