# Supplementary figures and images for: Production and purification of staphylococcal nuclease in Lactococcus lactis using a new expression-secretion system and a pH-regulated mini-reactor
Source: Microb Cell Fact. 2010 May 21;9:37. doi: 10.1186/1475-2859-9-37 (PMC2887397; doi:10.1186/1475-2859-9-37)

## Slide 1
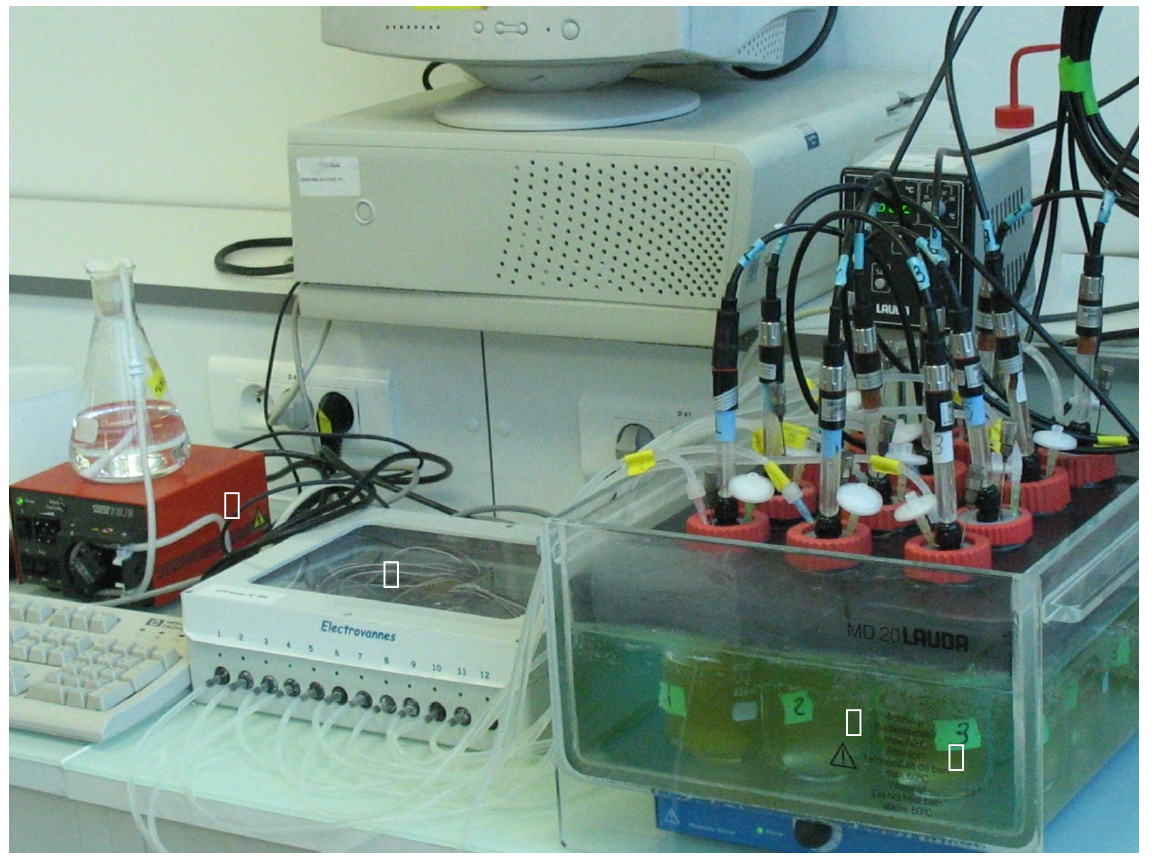






Supplement: Additional file 1 — LacMF, a parallel fermentation control system. 12 lactococcal cultures in mini-reactors of 200 mL can be made in parallel. They are maintained at 30°C and continously homogeneized by a magnetic stirrer ༉), and pH is controlled by supplying a neutralizing agent, NH4OH, via a proportional, integrative and derivative (PID) controller. NH4OH is added to the mini-reactors by a pump (⇐) with twelve solenoid valves (⇓). [file 1475-2859-9-37-S1.PPT]
